# Dyspareunia and pelvic pain: comparison of mid-urethral sling methods 10 years after insertion

**DOI:** 10.1007/s00192-023-05585-3

**Published:** 2023-07-10

**Authors:** Anna Lundmark Drca, Vasileios Alexandridis, Maria Andrada Hamer, Pia Teleman, Marie Westergren Söderberg, Marion Ek

**Affiliations:** 1 Department of Clinical Science and Education, Karolinska Institutet, Stockholm, Sweden; 2 Department of Obstetrics and Gynecology, Södersjukhuset, Stockholm, Sweden; 3 Department of Clinical Sciences Lund, Lund University, Lund, Sweden; 4 Department of Obstetrics and Gynecology, Skåne University Hospital, Lund, Sweden

**Keywords:** Dyspareunia, Mid urethral sling, Pelvic pain, Sexual dysfunction, Stress urinary incontinence

## Abstract

**Introduction and hypothesis:**

The mid-urethral sling (MUS) has been used for more than 30 years to cure stress urinary incontinence. The objective of this study was to assess whether surgical technique affects the outcome after more than ten years, regarding dyspareunia and pelvic pain.

**Methods:**

In this longitudinal cohort study we used the Swedish National Quality Register of Gynecological Surgery to identify women who underwent MUS surgery in the period 2006–2010. Out of 4348 eligible women, 2555 (59%) responded to the questionnaire sent out in 2020–2021. The two main surgical techniques, the retropubic and the obturatoric approach, were represented by 1562 and 859 women respectively. The Urogenital Distress Inventory-6 (UDI-6) and the Pelvic Organ Prolapse/Urinary Incontinence Sexual Questionnaire (PISQ-12), as well as general questions concerning the MUS surgery, were sent out to the study population. Dyspareunia and pelvic pain were defined as primary outcomes. Secondary outcomes included PISQ-12, general satisfaction, and self-reported problems due to sling insertion.

**Results:**

A total of 2421 women were included in the analysis. Among these, 71% responded to questions regarding dyspareunia and 77% responded to questions regarding pelvic pain. In a multivariate logistic regression analysis of the primary outcomes, we found no difference in reported dyspareunia (15% vs 17%, odds ratio (OR) 1.1, 95% CI 0.8–1.5) or in reported pelvic pain (17% vs 18%, OR 1.0, 95% CI 0.8–1.3) between the retropubic and obturatoric techniques among study responders.

**Conclusion:**

Dyspareunia and pelvic pain 10–14 years after insertion of a MUS do not differ with respect to surgical technique.

## Introduction

Stress urinary incontinence (SUI) is a large concern for many women [1]. It is mainly caused by damage to the supporting tissue of the urethra, and leads to urine leakage during increased intra-abdominal pressure [2].

SUI has been treated with minimally invasive surgery since the late 1990s. A mid-urethral sling (MUS), made of polypropylene mesh, is inserted to recover the support of the urethra. The mesh is non-absorbable and remains in the body as a permanent implant. This has become the gold standard treatment, and in Sweden 4000 surgeries per year are being performed. The retropubic and obturatoric approaches are the dominant techniques, and both have showed higher cure rates than pelvic floor muscle training and bulking agents in medium- and long-term outcomes [3–5]. The retropubic sling passes under the mid-urethra and then runs behind the pubic bone, whereas the obturatoric sling passes under the mid-urethra and then runs through the foramen obturatorium. More than 10 million women worldwide have had a MUS inserted due to SUI [6].

Pain in the genital and pelvic region after MUS is disputed, yet no definite benefit for any of the treatment methods has been established concerning sexual function [7–10]. During the last few years, insertion of slings has been debated. The use of this surgical method has decreased in many countries due to lobbying and reports concerning negative side effects. Some countries have even withdrawn the slings from the market [6]. The aim of this study was to assess dyspareunia and pelvic pain after MUS surgery and to investigate if there is any difference between the two main surgical techniques in long-term follow-up.

## Materials and methods

The study is a prospective cohort study. All Swedish women who underwent MUS surgery between 2006 and 2010 and had been registered in the Swedish National Quality Register of Gynecological Surgery (Gynop) received a postal questionnaire 10 to 14 years after insertion of a MUS. The Gynop register was initiated in 1997 and the majority of Sweden`s gynecological care institutions report to the register; coverage today is at least 85%. In the register we find information about pre- and postoperative symptoms, perioperative outcome, postoperative complications during the first year, and consequences of this such as re-operations and readmissions. Women report subjective symptoms and problems during the first post-operative year.

The questionnaire sent out included: Urogenital Distress Inventory (UDI-6) used to study urinary symptoms and impact on quality of life (the higher the score, the higher the disability), Incontinence Impact Questionnaire (IIQ-7) assessing impact of incontinence of life parameters (the higher the score the higher the impact), and Pelvic Organ Prolapse/Urinary Incontinence Sexual Questionnaire (PISQ-12), used to assess sexual function in women with urinary and prolapse symptoms. The maximum score (of the PISQ-12) is 48, higher scores indicating better sexual function [11, 12]. No exact cut-off level for good sexual function has been established. Demographic data and general questions about female health and the MUS surgery were included in the questionnaire. The primary outcome (dyspareunia) was assessed with the question: "Do you feel pain during intercourse?" The responders could answer “yes", or "no", or "do not know/do not want to answer”. The outcome pelvic pain was assessed according to question number six in UDI-6: "Do you experience pain or discomfort in your lower abdominal, pelvic, or genital area?" The responders could answer “yes" or "no", and then specify the level; "not at all", "a little bit", "moderately" or "greatly”. The secondary outcome was self-reported result after MUS surgery. It was measured by the question: "What do you think about the surgical outcome?" My condition is: “much improved", "improved", "unchanged", "deteriorated or "much deteriorated”. Consent was assumed if the questionnaire was returned by mail or if the weblink was used for reply.

Demographic data is presented as mean and standard deviation for continuous variables and as frequencies for categorical variables. When comparing variables between groups, we used the *t*-test and the Mann–Whitney U-test for continuous variables and the Chi-square test for categorical variables. *P*-levels < 0.05 were considered significant. The categorical endpoints,— dyspareunia, pelvic pain, and self-reported result after > 10 years, were analyzed in univariate and multivariate logistic regression. We used potential predictors of our endpoints in the regression models when assessing the effect of age, body mass index (BMI), childbirth, smoking, menopausal status, menopausal hormone treatment (MHT) and type of MUS. Results from the regression analyses are presented both as crude and adjusted odds ratios with confidence intervals of 95%. Statistical analyses were performed using IBM SPSS Statistics 28. The study was approved by the Ethical Review Board at Karolinska Institutet, Stockholm dnr 2019–02529.

## Results

**Fig. 1:**
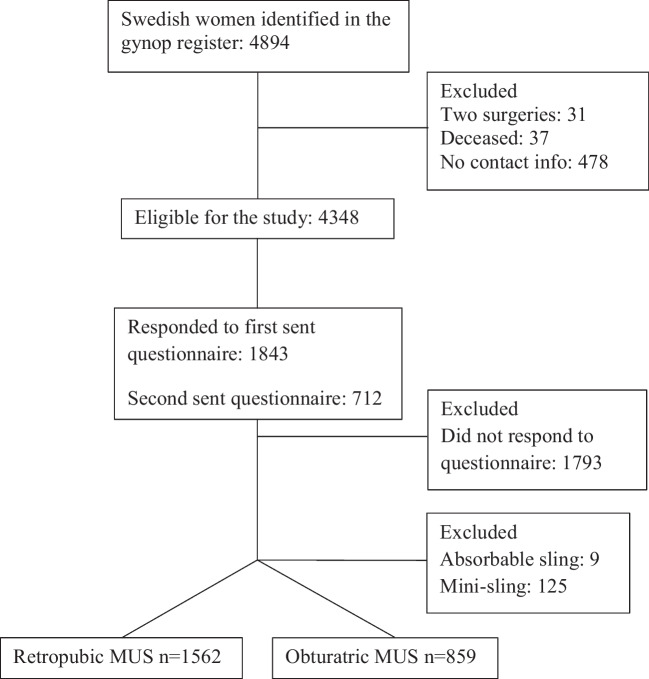
Flowchart of the study population

Figure 1 shows a flowchart of the study population. A total of 4348 women were eligible, and 2555 (59%) women responded to the questionnaire. Out of these, 1562 women had received a retropubic sling and 859 an obturatoric sling. We excluded women who had obtained an absorbable sling or a single incision sling (mini-sling) (*n* = 134). Baseline data of the study-groups is summarized in Table 1.

**Table 1:** Demographic and clinical characteristics of the study population

	Retropubic (*n* = 1562)	Obturatoric (*n* = 859)
Age at MUS surgery (years)	52.9 (± 11.1)	53.6 (± 10.7)
Age at follow-up (years)	64.2 (± 11.1)	65.0 (± 10.7)
Years after MUS surgery	11.3 (± 0.8)	11.4 (± 0.8)
BMI at MUS surgery	26.2 (± 5.8)	26.8 (± 6.6)
BMI at follow-up	26.7 (± 8.2)	27.1 (± 4.7)
BMI ≥ 30 at surgery	235 (15)	149 (20)
BMI ≥ 30 at follow-up	285 (18)	184 (22)
Smokers	108 (7)	60 (7)
Childbirth	1470 (96)	800 (95)
Postmenop at follow-up	1261 (91)	717 (92)
MHT	655 (44)	385 (47)

MUS = midurethral sling; BMI = body mass index; postmenop = postmenopausal; MHT = menopausal hormone treatment (local or systemic oestrogen)

Data are mean (± standard deviation) or *n* (%)

**Fig. 2:**
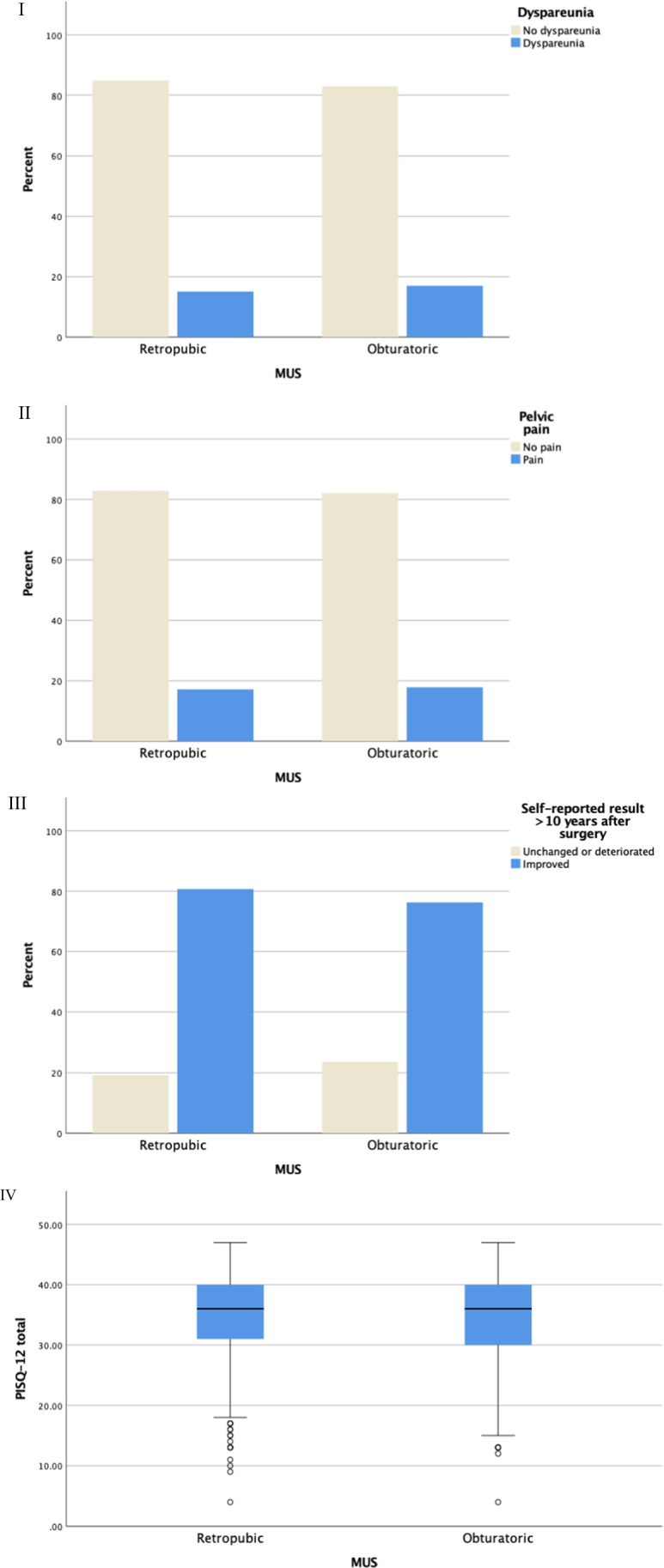
**I** Dyspareunia with respect to MUS technique. **II** Pelvic pain with respect to MUS technique. **III** Self-reported result with respect to MUS technique. **IV **PISQ-12 with respect to MUS technique

When comparing the two MUS techniques with regard to dyspareunia there was no significant difference. A total of 1685 women (70%) answered the question “Do you have pain during intercourse?” Out of those, 165 (15%) in the retropubic group reported dyspareunia and 101 (17%) in the obturatoric group [OR 1.2 (0.8–1.5) *p* = 0.3] (Fig. 2).

Neither was there any difference between the retropubic and obturatoric MUS with regard to pelvic pain. Seventeen and 18% respectively admitted suffering from this [OR 1.1 (0.8–1.5) *p* = 0.7]. The question being asked was according to UDI-6 “Do you experience pain or discomfort in your lower abdominal, pelvic, or genital area?” (Fig. 2:I). In addition, we investigated sexual function for the study groups with PISQ-12. In total, 1264 women (52%) responded to the questionnaire. Mean age for those who responded was 60 years (± 9) ,whereas the mean age for non-responders was 70 years (± 11). We observed no difference between the two surgical groups regarding PISQ-12, with a mean value of 34.9 (± 6.8) for the retropubic technique and 34.4 (± 7.3) for the obturatoric technique (*p* = 0.39) (Fig. 2:IV). Dyspareunia according to PISQ-12 did not differ between the two groups [OR 1.1 (0.9–1.4) *p* = 0.47]. Leakage of urine during sex is assessed in PISQ-12, and here we found 114 (13%) in the retropubic group and 79 (17%) (*p* = 0.05) in the obturatoric group suffering from this always, often, or sometimes.

The secondary outcome "treatment success” — defined as “What do you think about the surgical outcome?” — was answered by 2365 women (98%) in total. In the retropubic group, 1234 (81%) and in the obturatoric group 639 (76%) agreed that they were in a better or much better condition than before the surgery (*p* = 0.011) (Fig. 2:III). We also posed the question: “Have you had any problems connected to the sling inserted?”; the response rate was 96%. In the retropubic group, 35 (2.3%) participants, and 25 (3.1%) in the obturatoric group reported suffering from tightness of the vagina (*p* = 0.28). Furthermore, 53 (3.5%) women in the retropubic group and 33 (4.0%) in the obturatoric group reported pain due to the sling (*p* = 0.52). Finally, dyspareunia directly connected to the sling was reported by 78 (5.2%) and 49 (6.0%) individuals respectively (*p* = 0.28).

We performed a logistic regression analysis to further examine risk factors and clinical characteristics for dyspareunia, pelvic pain, and unsuccessful treatment. In the multivariate logistic regression, we adjusted for age and BMI at baseline, childbirth, current smoking, menopausal state, MHT, and type of MUS inserted. Table 2 presents the number of complete cases analyzed in the multivariable analysis. Factors associated with dyspareunia were shown to be age at surgery (*p* for trend = 0.004, with less dyspareunia with increasing age), BMI at surgery (*p* for trend = 0.008, with increasing dyspareunia with higher BMI), but also menopausal state at follow-up [OR 2.0 (95% CI 1.0–3.8) *p* = 0.04] and use of MHT at follow-up [OR 2.1 (95% CI 1.5–2.8), *p*  < 0.001]. The type of MUS was not a significant predictor [OR 1.1 (95% CI 0.8–1.5) *p* = 0.54]; Table 3.

**Table 2:** Missing data for primary and secondary outcomes and complete cases for multivariate logistic regression

	Missing data	Missing data	Complete cases: multivariate analysis*	Complete cases: multivariate analysis*
Outcome	Retropubic	Obturatoric	Retropubic	Obturatoric
Dyspareunia	470 (30)	266 (31)	859 (55)	467 (54)
Pelvic pain	26 (2)	21 (2)	1174 (75)	634(74)
Dyspareunia (PISQ-12)	686 (44)	396 (46)		
PISQ-12	749 (48)	408 (47)		
Result	34 (2)	22 (3)	1181 (76)	648 (75)

^*^ = multivariate logistic regression analysis for risk factors concerning dyspareunia, pelvic pain, and self-reported result

Data is shown as numbers (%)

**Table 3:** Odds ratio of dyspareunia, pelvic pain and self-reported result > 10 years after MUS surgery by baseline characteristics and clinical covariates

	Dyspareunia		Pelvic pain		Self-reported improvement > 10 years after surgery	
	OR (95% CI)	aOR (95% CI)	OR (95% CI)	aOR (95% CI)	OR (95% CI)	aOR (95% CI)
Age at surgery (years)						
< 50	Referent	Referent	Referent	Referent	Referent	Referent
50–59	1.3 (1.0–1.8)	1.0 (0.7–1.5)	0.9 (0.7–1.2)	0.7 (0.5–0.9)	0.6 (0.5–0.8)	0.8 (0.6–1.0)
60–69	0.8 (0.5–1.1)	0.5 (0.3–0.8)	1.1 (0.8–1.4)	0.8 (0.5–1.1)	0.4 (0.3–0.5)	0.5 (0.3–0.7)
≥ 70	0.2 (0.1–0.9)	0.1 (0.01–0.7)	0.8 (0.5–1.3)	0.5 (0.3–0.9)	0.3 (0.2–0.4)	0.3 (0.2–0.5)
*P* for trend	0.006*	0.004*	0.63	0.03*	< 0.001*	< 0.001*
BMI at surgery						
< 25	Referent	Referent	Referent	Referent	Referent	
25 to < 30	0.8 (0.5–1.0)	0.7 (0.5–1.0)	1.5 (1.1–1.9)	1.5 (1.2–2.0)	0.6 (0.5–0.8)	0.6 (0.5–0.8)
≥ 30	1.3 (0.9–1.9)	1.4 (1.0–2.1)	2.4 (1.8–3.2)	2.5 (1.8–3.6)	0.3 (0.2–0.4)	0.4 (0.3–0.5)
*P* for trend	0.03*	0.008*	< 0.001	< 0.001*	< 0.001*	< 0.001*
Childbirth						
Yes	0.7 (0.4–1.4)	0.6 (0.3–1.2)	0.9 (0.5–1.4)	1.1 (0.6–2.1)	1.8 (1.1–2.8)	1.4(0.8–2.4)
No	Referent	Referent	Referent	Referent	Referent	Referent
*P*	0.31	0.13	0.59	0.69	0.01*	0.24
Smoking						
Yes	1.7 (1.1–2.6)	1.5(0.9–2.5)	1.43(0.9–1.9)	1.2 (0.8–2.0	0.8 (0.5–1.1)	0.9 (0.5–1.4)
No	Referent	Referent	Referent	Referent	Referent	Referemt
*P*	0.02*	0.16	0.18	0.19	0.22	0.54
Postmenopausal						
Yes	2.3 (1.3–4.1)	2.0 (1.0–3.8)	1.1 (0.7–1.6)	1.0 (0.6–1.6)	0.6 (0.4–0.9)	1.0 (0.6–1.6)
No	Referent	Referent	Referent	Referent	Referent	Referent
*P*	0.003*	0.04*	0.70	1.0	0.02	0.95
MHT						
Yes	2.1 (1.6–2.8)	2.1(1.5–2.8)	1.7 (1.4–2.1)	1.9 (1.5–2.5)	0.8 (0.6–0.9)	0.8 (0.6–1.0)
No	Referent	Referent	Referent	Referent	Referent	Referent
*P*		< 0.001*		< 0.001*	0.01*	0.005*
MUS						
Retropubic	Referent	Referent	Referent	Referent	Referent	Referent
Obturatoric	1.2 (0.9–1.5)	1.1 (0.8–1.5)	1.1 (0.8–1.3)	1.0 (0.8–1.3)	0.8 ( 0.6–0.9)	0.8 (0.6–1.0)
*P*	0.30	0.54	0.66	1.0	0.02*	0.03*

OR, odds ratio; aOR, adjusted odds ratio; BMI, body mass index; MHT, menopausal hormone treatment (oestrogen supplement locally or systemically); MUS, mid urethral sling. Data is *n* (%) or OR (95% CI)

Results categorized for age at surgery, BMI at surgery, childbirth, smoking at follow-up, menopausal state at follow-up, MHT at follow-up, MUS

The primary outcome pelvic pain was associated with age at surgery (*p* for trend = 0.03), BMI at surgery (*p* for trend < 0.001) and use of MHT at follow-up (OR 1.9 (95% CI 1.5–2.5) *p*  < 0.001). Furthermore, we found that the type of MUS inserted was not associated [OR 1.0 (95% CI 0.8–1.3) *p* = 1.0) with pelvic pain (Table 3).

## Discussion

We found that dyspareunia and pelvic pain 10–14 years after insertion of a MUS do not differ with respect to the surgical technique used. We found that sexual function according to PISQ-12 does not vary between the study groups. Most women in both groups also expressed a general improvement that persisted.

To our knowledge there are no studies that have investigated the situation concerning sexual function and pain a long time after MUS surgery. Earlier studies, however, show divergent results concerning pain a short time after surgery. The obturatoric MUS is thought to increase the risk of dyspareunia [13] and early postoperative and groin pain [5, 14, 15]. We know that the innervation of the clitoris may be affected by insertion of an MUS, and thus risk affecting the orgasmic function [16]. Additionally, slings inserted retropubically can affect the blood flow of the clitoris and are thought to cause a higher risk of damage of the dorsal nerve of the clitoris [17, 18]. Nevertheless, our study did not reveal any long-term difference with regard to dyspareunia, pelvic pain, or sexual function between the two methods. A literature review from 2012 on sexual function after SUI surgery reported 88% of women having an unchanged or improved sexual function [19]. Our study shows that a general improvement remains for a majority of women in the long term, since 76 to 81% of our study participants still reported general improvement after 10 years. Interestingly, we found a more than doubled risk of suffering from pelvic pain and an almost three times higher risk of suffering from dyspareunia in the group reporting no general improvement after surgery compared to those reporting improvement. The cause of this increased risk is unclear. However, it may be assumed that the presence of dyspareunia and pelvic pain would affect one’s notion of improvement after the surgical intervention. The cohort taken from the Gynop register is a big group that well represents elderly women from different backgrounds and geographic areas in Sweden. The register is prospective, which eliminates recall-bias concerning surgical technique. We believe therefore that the generalizability is high. Other strengths of the study are that it combines register data with self-reported data and that almost 60% of the eligible women were included in the study. We have no reason to believe that dropout is larger in any of the groups. According to the dropout analysis, 1065 (63%) of the non-responders went through retropubic MUS and 622 (37%) obturatoric MUS, compared with responders — 1562 (65%) versus 859 (36%). Still, missing information from 41% of the study group can of course influence the results and must be considered as a limitation. We know that age of the dropouts is somewhat higher than for the responders with a mean age of 68 (± 13) years. This, we believe, is partly due to decreasing likelihood of being sexually active with increasing age, and that other problems and diseases override. Concerning sexual activity among responders, we have to assume that women responding to the question about dyspareunia are sexually active. Questions about being sexuallay active or not are lacking in all questionnaires used in this study, both the pre-operative questionnaire sent out by the Gynop register and also the 10-year follow-up, and this is of course a limitiation. With regard to the prevalence of dyspareunia among women in general, our results do not differ from earlier studies that have showed a prevalence among women ranging from 7.5 to 43% [20–23], although women in our follow-up are older than women in earlier studies. In our study, 10% of women who underwent retropubic MUS surgery reported dyspareunia preoperatively, compared with 13% at 1 year postoperatively and 15% at 10 years postoperatively. The corresponding results for the obturatoric MUS were 12%, 14%, and 17% respectively. The response rates were 52% preoperatively, 35% after 1 year, and 70% after 10 years or more. This indicates however a slow progression of dyspareunia in 10 years, but no difference found between surgery groups.

Over 50% of the women studied reported PISQ-12 points over 35. Since no cut-offs exist for PISQ-12, and sexual function of elderly women is scarcely studied, we cannot relate our study participants´ scores. However, we believe that the scores reported are quite high for the age group represented in the study (Fig. 2:IV).

Coital incontinence seems to be somewhat higher in the obturatoric group than in the retropubic group and could possibly explain why in total the retropubic group reports being more satisfied. Cure of coital incontinence has previously been described as an important predictor concerning sexual function after MUS surgery [24, 25].

In conclusion, we found no difference at long-term follow-up between the retropubic technique and the obturatoric technique with regard to dyspareunia and pelvic pain. Still, a small difference between the reported improvement after more than 10 years shows a small advantage for the retropubic technique. Sexual function according to PISQ-12 was not found to differ between the studied groups.

To further investigate the long-term safety of MUS slings in term of pain and sexual function, a comparison between our study groups and age-matched controls would be beneficial. The long-term effect and safety of MUS surgery must be put in focus and make it possible for women to choose a favourable treatment for SUI.
